# Aquaporin-4 prevents exaggerated astrocytosis and structural damage in retinal inflammation

**DOI:** 10.1007/s00109-022-02202-6

**Published:** 2022-05-10

**Authors:** Ali Maisam Afzali, Lasse Stüve, Monika Pfaller, Lilian Aly, Katja Steiger, Benjamin Knier, Thomas Korn

**Affiliations:** 1 Institute for Experimental Neuroimmunology, Technical University of Munich School of Medicine, Munich, Germany; 2 Department of Neurology, Technical University of Munich School of Medicine, Munich, Germany; 3 Munich Cluster for Systems Neurology (SyNergy), Munich, Germany; 4 Institute of Pathology, Technical University of Munich School of Medicine, Munich, Germany

**Keywords:** AQP4, EAE, Retina, GFAP, Astrocytosis, OCT, OCT angiography

## Abstract

**Abstract:**

Aquaporin-4 (AQP4) is the molecular target of the immune response in neuromyelitis optica (NMO) that leads to severe structural damage in the central nervous system (CNS) and in the retina. Conversely, AQP4 might be upregulated in astrocytes as a compensatory event in multiple sclerosis. Thus, the functional relevance of AQP4 in neuroinflammation needs to be defined. Here, we tested the role of AQP4 in the retina in MOG(35–55)-induced experimental autoimmune encephalomyelitis (EAE) using optical coherence tomography (OCT), OCT angiography, immunohistology, flow cytometry, and gene expression analysis in wild-type and *Aqp4*^–/–^ mice. No direct infiltrates of inflammatory cells were detected in the retina. Yet, early retinal expression of TNF and Iba1 suggested that the retina participated in the inflammatory response during EAE in a similar way in wild-type and *Aqp4*^–/–^ mice. While wild-type mice rapidly cleared retinal swelling, *Aqp4*^–/–^ animals exhibited a sustainedly increased retinal thickness associated with retinal hyperperfusion, albumin extravasation, and upregulation of GFAP as a hallmark of retinal scarring at later stages of EAE. Eventually, the loss of retinal ganglion cells was higher in *Aqp4*^–/–^ mice than in wild-type mice. Therefore, AQP4 expression might be critical for retinal Müller cells to clear the interstitial space from excess vasogenic edema and prevent maladaptive scarring in the retina during remote inflammatory processes of the CNS.

****Key messages**:**

- Genetic ablation of AQP4 leads to a functional derangement of the retinal gliovascular unit with retinal hyperperfusion during autoimmune CNS inflammation.
- Genetic ablation of AQP4 results in a structural impairment of the blood retina barrier with extravasation of albumin during autoimmune CNS inflammation.
- Eventually, the lack of AQP4 in the retina during an inflammatory event prompts the exaggerated upregulation of GFAP as a hallmark of scarring as well as loss of retinal ganglion cells.

**Supplementary Information:**

The online version contains supplementary material available at 10.1007/s00109-022-02202-6.

## Introduction

Objective assessment of inflammatory activity in neuroimmunologic diseases such as multiple sclerosis (MS) and neuromyelitis optica (NMO) is essential for selecting appropriate disease-modifying therapies. Optical coherence tomography (OCT) offers a noninvasive approach to longitudinally measure retinal changes, which can be used as a surrogate for inflammatory activity in the central nervous system (CNS). Notably, changes in certain retinal layers such as the peripapillary retinal nerve fiber layer (pRNFL) or the inner nuclear layer (INL) have been correlated with clinical and magnetic resonance imaging (MRI) parameters in MS [1–3]. These observations reinvigorated attempt to understand retinal pathology during neuroinflammatory diseases. Here, we focused on the role of the water channel protein aquaporin-4 (AQP4), which is expressed in retinal radial glia (Müller cells). AQP4 is a direct and may be an indirect target of the immune responses in NMO and MS, respectively [4]. However, the functional relevance of AQP4 expression in the retina is only incompletely understood.

At least two different processes are being discussed as drivers of retinal pathology in MS and NMO [5–7]. On the one hand, the term “retrograde maculopathy” was introduced as retrograde axonal degeneration resulting from lesions in the anterior visual pathways [8]. On the other hand, changes in retinal thickness have been associated with direct changes in retinal fluid homeostasis during inflammatory processes [5, 9]. In the CNS, AQP4 is expressed in astrocytes. Astrocytic AQP4 might be required to maintain the hydrostatic flow of solutes and particles through the interstitial space via the cerebrospinal fluid (CSF) to the venous limb of the circulation and thus be an integral component of the glymphatic system [6]. Consequently, loss of function of AQP4 or impaired redistribution of the channel to the plasma membrane may result in reduced flow through the glymphatic system [10, 11]. In line with this concept, loss of function of AQP4 leads to worse outcome in disease conditions associated with vasogenic edema such as brain tumor or brain abscess [11] but protects from cytotoxic edema, e.g., in the context of brain ischemia [12]. A similar system of flow through the interstitial space has been proposed for the retina, which might be mediated by AQP4 expressed in Müller cells [5]. Therefore, we raised the hypothesis that Müller cells (through their expression of AQP4) might be an important component of resilience to inflammatory stress in the retina.

Since recent work by us and others reproduced key retinal findings from studies on MS patients in experimental autoimmune encephalomyelitis (EAE) [9, 13], we explored the role of AQP4 in retinal pathology in the MOG(35–55)-induced EAE model, which involves the retina through innate immune activation [14]. We found that lack of AQP4 results in increased and sustained retinal thickness in longitudinal OCT analyses and enhanced albumin extravasation in *Aqp4*^–/–^ mice as compared with wild type control animals during EAE. Eventually, loss of AQP4 in the retina led to increased astrogliosis and scarring with exaggerated loss of retinal ganglion cells, which might fix a dysfunctional state of the retina in *Aqp4*^–/–^ mice even after the inflammation is cleared. In summary, these observations support a protective functional role of AQP4 in the retina in autoimmune CNS diseases.

## Methods

### Mice

C57BL/6 J mice were initially obtained from Jackson Laboratories and bred in our facility. *Aqp4*^–/–^ mice were kindly provided by A. Verkman (University of California, San Francisco UCSF) and have been described before [12]. All mice were housed in a pathogen‐free facility at the Technical University of Munich. All experimental protocols were approved by the standing committee for experimentation with laboratory animals of the Bavarian state authorities and carried out in accordance with the corresponding guidelines (ROB-55.2–2532.Vet_02-13–29, ROB-55.2–2532.Vet_02-17–69, ROB-55.2–2532.Vet_02-17–234, ROB-55.2–2532.Vet_03-18–53, ROB-55.2–2532.Vet_02-21–140, ROB-55.2–2532.Vet_02-21–154).

### EAE induction

Mice were immunized subcutaneously at the base of tail with 200 μl of an emulsion containing 200 μg of MOG peptide (35–55), MEVGWYRSPFSRVVHLYRNGK (Auspep, Tullamarine, Australia) and 250 μg *Mycobacterium tuberculosis* H37Ra (BD Difco) in mineral oil (CFA). Sham controls were immunized with PBS in CFA instead of MOG(35–55). In addition, mice received 200 ng pertussis toxin (Ptx, Sigma, Cat# P7208) i.v. on days 0 and 2 after immunization. Clinical signs of disease were monitored daily with scores as follows: 0, no disease; 1, loss of tail tone; 2, impaired righting; 3, paralysis of both hind limbs; 4, tetraplegia; 5, moribund state [15].

### OCT/OCT-A analysis

Murine OCT images were acquired as previously described [16] using a commercially available spectral-domain OCT device (Heidelberg Engineering Spectralis OCT 2). Briefly, mice were anesthetized by i.p. application of medetomidine (0.5 mg/kg), midazolam (5 mg/kg), and fentanyl (0.05 mg/kg); eyes were treated with eye drops containing 2% atropine before placing a 100‐diopter contact lens (Roland Consult, Germany) with contact gel on the eye to be examined. For adaptation to murine eyes, a 78‐diopter lens (double aspheric Volk lens 78D; Volk Optical Inc., Mentor, USA) was installed directly in front of the OCT machine. The retina was analyzed using a volume scan manually centered on the optic nerve head (ONH) consisting of 49 vertical B‐scans with a scanning angle of 20° × 20°. Both eyes were measured and each scan was assessed for sufficient signal strength (> 20 dB), adequate illumination, and accurate beam placement. Only scans that met these quality criteria were used for analysis. All B‐scans were segmented automatically into different layers using a software-inbuilt algorithm (Heyex PACS, Eye Explorer, v2.5.4.), checked manually, and corrected if necessary in a blinded manner as per the APOSTEL 2.0 recommendations [17]. The ONH was identified through the opening of Bruchs membrane and excluded for volumetric analyses. For longitudinal measurements, a company‐derived “follow‐up” software mode was used, which enabled repetitive analyses of the exact same place.

Retinal perfusion was analyzed using OCT angiography (OCT-A) at the same spectral-domain OCT with angiography module (Heidelberg Engineering). Consecutive scans at one location of the retina were acquired, and after removal of stationary tissue signals, the remaining signal reflected the area-intrinsic motion of corpuscular blood constituents in both venous and arterial blood vessels. For OCT-A imaging, en face images and decorrelation signals were recorded with a 10° × 10° angle and a lateral resolution of 5.7 μm/pixel, resulting in a 2.5 × 2.5 mm area focusing on the ONH. A full-spectrum amplitude decorrelation algorithm was employed for motion detection and image creation, and active eye tracking was accomplished by TruTrack. Segmentation of the ONH area was performed automatically into the superficial vascular complex (SVC) and the deep vascular complex (DVC) by the in-built software (v2.5.4) and, if necessary, corrected manually in a blinded manner. The vessel density was defined as the proportion of the detected vessel pixels in an extracted OCT-A image (total area 512 × 512 pixels) and determined using ImageJ software (v.1.53 k, RRID:SCR_003070). Changes in vessel density were quantified in relation to the vessel density on d0 (Δratio vessel density = 1 – dx/d0). Images with obvious problems, decentration of the imaging focus, and major motion artifacts were excluded. Again, we excluded the ONH for quantitative analysis.

Both OCT and OCT-A measurements were performed longitudinally prior to immunization (baseline, d0), d15 post immunization (p.i.), d32 p.i., and d50 p.i. To quantify the change in retinal thickness during EAE, we calculated the difference between each follow-up time and d0 (Δthickness = dx – d0).

### Quantitative PCR

Total RNA was isolated from the whole retina with RNA-easy Plus micro kit (Qiagen, Cat# 74,034) after mechanical disruption using TissueRuptor (Qiagen). The isolated RNA was transcribed into cDNA using the TaqMan Reverse Transcription Reagents Kit (Life Technologies, Cat# N8080234) according to the manufacturer’s instructions. Probes were purchased from Life Technologies and the assays were performed on 96-well reaction plates (Life Technologies). The real-time PCR was performed on StepOnePlus system (Life Technologies). In all experiments, β-actin was used as reference gene to calibrate gene expression.

## Histology

Mice were sacrificed under deep anesthesia by intracardial perfusion with PBS followed by perfusion with 4% w/v paraformaldehyde (PFA) solved in PBS. Brains, optic nerves, eyes, kidneys, and spleens were removed and fixed in 4% PFA overnight. Vertebral columns including the spinal cords were additionally decalcified with OSTEOSOFT® (Sigma-Aldrich, Cat# 1,017,281,000) for 72 h prior to paraffin embedding; 2 μm thick sections were prepared. Immunohistochemistry was performed using a Leica Bond Rxm System with a Polymer Refine detection kit (Leica, Cat# DS9390). CD45 (Thermo, 30-F11, Cat# 14–0451-82, 1:200), GFAP (Sigma, G-A-5, Cat# G6171, 1:500), glutamine-synthetase (BD, 6/Glutamine Synthetase, Cat# ab49873, 1:15.000), Iba1 (Wako, #019–19,741, 1:500), and albumin (Thermo, JF32-1a, Cat# MA5-32,531, 1:200) were detected with primary antibodies raised in rat. As secondary antibody, a rabbit anti rat antibody (Vector AI-4001, 1:400) was used. DAB was used as chromogen, and counterstaining was performed with hematoxylin. Albumin staining was performed without counterstaining where indicated. The slides were then scanned on a Leica AT2 system, and the images were analyzed using Aperio Image Scope (Leica) in a blinded manner.

Quantification of histological samples was performed automatically with computer-assisted algorithms provided by QuPath v0.3.2 software (https://qupath.github.io, University of Edinburgh, Scotland). For detection of total counts of retinal ganglion cells (RGC), ganglion cell layer (GCL) of the whole retina section was annotated and analyzed automatically by positive nuclear detection. Albumin stainings were determined with positive pixel counts after annotation of whole retinae and quantified as mean positive percentage of total region of interest (ROI). For GFAP analysis, the inner plexiform layer (IPL) and the inner nuclear layer (INL) were annotated separately. All annotations were performed in a blinded manner.

### Isolation of mononuclear cells from CNS

At defined time points, CNS-infiltrating cells were isolated after perfusion through the left cardiac ventricle with PBS. The brain with attached optic nerves and the spinal cord were extracted and digested with collagenase D (2.5 mg/ml) and DNase I (1 mg/ml) at 37 °C for 45 min. After passing the tissue through a 70-μm cell strainer, cells of the spinal cord and brain were separated by discontinuous Percoll gradient (70%/37%) centrifugation. Mononuclear cells were isolated from the interphase for further analyses (flow cytometry).

### Intracellular Cytokine Staining and Flow Cytometry

Mononuclear cells were stained with LIVE/DEAD fixable dyes (Aqua [405 nm excitation], Invitrogen, Cat# L34966), and antibodies to surface markers: CD3e (145-2C11), CD4 (GK1.5 or RM4-5), CD8a (53–6.7), CD11b (M1/70), CD11c (HL3), CD19 (1D3 or 6D5), CD45 (30-F11), all BD Biosciences, eBioscience, or BioLegend. For intracellular cytokine staining, cells were stimulated ex vivo with 50 ng/ml PMA (Sigma-Aldrich, Cat# P1585), 1 μg/ml ionomycin (Sigma-Aldrich, Cat# 10,634), and monensin (1 μl/ml BD GolgiStop, Cat# 554,724) at 37 °C for 2.5 h. Subsequent to LIVE/DEAD and surface staining, cells were fixed and permeabilized (Cytofix/Cytoperm and Perm/Wash Buffer; BD Biosciences, Cat# 554,722 and 554,723) and stained for Foxp3 (FJK-16 s) and the cytokines IL-17A (TC11-18H10.1; BioLegend) and IFN-γ (XMG1.2; eBioscience). Cells were analyzed using a CytoFlex S (Beckman Coulter). Data analysis was performed with FlowJo version 10 (Tree Star, RRID:SCR_008520).

## Statistical analysis

In all experiments, both retinae from one mouse were processed individually, and the mean of both eyes was used for quantification. Statistical evaluations of cell numbers, MFIs, and OCT data were performed using unpaired student’s *t* tests when two populations were compared. Two‐tailed *p* values < 0.05 were considered significant. Multiple comparisons were performed with two‐way ANOVA followed by specific post‐tests as indicated in the legends to the figures. Fisher’s exact test was used to compare EAE incidences. Age at immunization, day of disease onset and peak EAE scores were compared using Mann–Whitney *U* tests. In some cases, the disease burden between strains was compared by linear regression analysis. Statistical analysis was performed using GraphPad Prism 8 software (version 8.4.3).

## Results

### *Aqp4*^–/–^ mice are susceptible to MOG(35–55) induced EAE

**Fig. 1:**
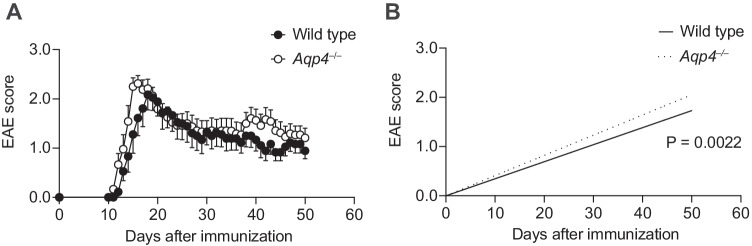
*Aqp4*^–/–^ mice are highly susceptible to active EAE. Active EAE was induced by immunization with MOG(35–55) in complete Freund's adjuvant (CFA) plus pertussis toxin in wild-type and *Aqp4*^–/–^ mice and monitored daily. Sham controls were immunized with an emulsion containing PBS instead of MOG(35–55) and showed no signs of disease (data not shown). **A** Mean EAE scores ± SEM of wild-type and *Aqp4*^–/–^ mice with clinical signs of EAE. **B** Linear regression analysis of disease scores of wild-type and *Aqp4*^–/–^ mice with clinical signs of EAE indicated a higher disease load in *Aqp4*^–/–^ mice than in wild type mice (*P* = 0.0022)

Astrocytic AQP4 is supposed to facilitate cytotoxic edema but protect from extracellular (vasogenic) edema due to its function in clearing the interstitial space from excess fluid load. We wanted to test the role of AQP4 in autoimmune tissue inflammation in the CNS, in which interstitial edema is an early hallmark of pathology. Therefore, we induced EAE through active immunization with MOG(35–55) in control wild-type and *Aqp4*^–/–^ mice. The disease incidence of *Aqp4*^–/–^ mice was higher than in wild-type animals (Table 1). Both strains developed classical EAE, and the overall disease burden of sick *Aqp4*^–/–^ mice was at least as high as that of sick wild-type animals (Fig. 1A, B).

**Table 1:** Incidence and severity of MOG(35–55)-induced EAE in wild-type and *Aqp4*^–/–^ mice

	**WT** **(Sham)**	***Aqp4***^**–/–**^ **(Sham)**	**WT**	***Aqp4***^**–/–**^	***P* value**
EAE incidence [%] (total)	0 (0/16)	0 (0/16)	56.6 (30/53)	86.3 (44/51)	0.002
Median age at imm. [weeks, IQR]	11.6 [8.0–12.0]	13.4 [9.6–14.7]	12.0 [7.9–16.3]	10.4 [9.6–13.1]	0.526
Mean day of onset (± SEM)	n.a	n.a	14.6 (± 0.8)	12.7 (± 0.4)	0.079
Mean peak EAE score (± SEM)	n.a	n.a	2.3 (± 0.25)	2.5 (± 0.16)	0.342

Clinical parameters and EAE characteristics of all mice in the respective groups including EAE incidence, age at immunization, day of disease onset, and peak EAE scores. IQR, interquartile range; p.i, post immunization. Fishers exact test was used to compare EAE incidences. Age at immunization, day of disease onset and peak EAE scores were compared using a Mann–Whitney *U* test, * *P* < 0.05

### *Aqp4*^–/–^ mice experience a sustained retinal thickening during recovery from EAE

**Fig. 2:**
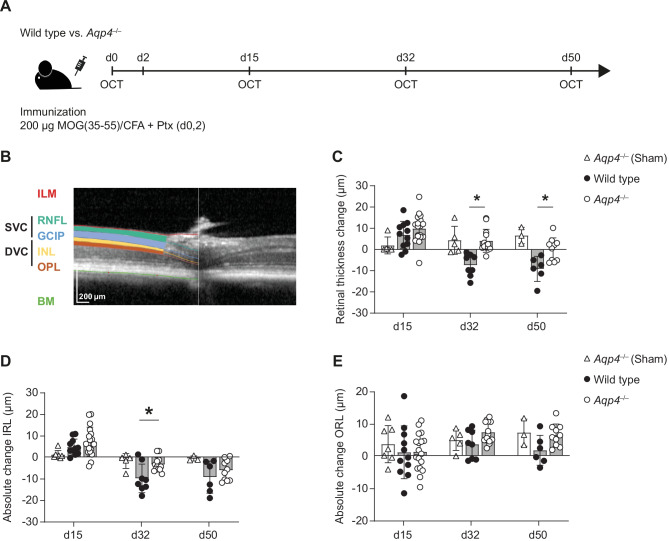
Longitudinal in vivo-optical coherence tomography (OCT) imaging of the retina during EAE. **A** Longitudinal OCT measurements were performed in immunized wild-type and *Aqp4*^–/–^ mice prior to immunization (baseline), on d15 p.i., d32 p.i., and d50 p.i. as indicated. **B** The retina was analyzed using a volume scan centered on the optic nerve head (ONH). Layers were segmented automatically with Eye Explorer software and corrected manually in a blinded manner as per APOSTEL 2.0 recommendations [17]. As defined in [17], IRL comprises retinal nerve fiber layer (RNFL), ganglion cell layer (GCL), and inner plexiform layer (IPL). ORL comprises layers between inner and outer limiting membrane. A representative segmentation of the retina is shown. The localization of the SVC and DVC are indicated. **C** Absolute change in retinal thickness over time during EAE. At baseline, wild-type and *Aqp4*^–/–^ mice did not differ in mean retinal thickness (wild-type mice 237.39 ± 7.98 µm vs. *Aqp4*^–/–^ mice 233.34 ± 5.44 µm). Mean absolute change in IRL (**D**) and ORL (**E**) thickness ± SD as compared to baseline. Significance in C-E was calculated using two-way ANOVA and Sidak’s post test. * *P* < 0.05. BM, Bruch’s membrane; DVC, deep vascular complex; ILM, inner limiting membrane; INL, inner nuclear layer; GCIP, combination of ganglion cell and inner plexiform layer; OPL, outer plexiform layer; RNFL, retinal nerve fiber layer; SVC, superficial vascular complex

Inherited retinal pathology has been described in some C57BL/6 strains. Even though we used C57BL/6 J mice (and not C57BL/6 N mice, in which the Rd8 mutation of the Crb1 gene has been reported [18]), we first excluded that our experimental animals did not carry the Rd8 mutation as a potential confounder of inflammatory retinal changes (Supplementary Fig. 1). Since we were interested in exploring OCT as a means to monitor retinal changes as a surrogate for tissue responses to inflammation in the CNS, we carried out longitudinal OCT measurements at defined time points after induction of EAE in wild-type and *Aqp4*^–/–^ mice (Fig. 2A). It is known that the retinal thickness of wild-type mice increases up to the peak of EAE (~ d15 p.i.) and then decreases below baseline levels in the recovery phase of EAE (> d21 p.i.) [9, 13]. This effect is mainly due to changes in the inner retinal layers (IRL) comprising the RNFL, ganglion cell layer (GCL), and inner plexiform layer [9, 13] (Fig. 2B). We were able to reproduce this phenomenon in our wild-type cohort (Fig. 2C, Supplementary Table 1). While retinal swelling at the peak of EAE was comparable between wild-type and *Aqp4*^–/–^ mice, the decline in retinal thickness after the peak of EAE was significantly delayed in *Aqp4*^–/–^ mice as compared to wild-type animals. Here, retinal thickness measures of *Aqp4*^–/–^ mice reached baseline levels only on day 50 after immunization (Fig. 2C, Supplementary Table 1). Differential responses in wild-type vs *Aqp4*^–/–^ retinae in EAE appeared to be mainly driven by alterations of the inner retinal layers (Fig. 2D, E), which is in line with findings in humans in MS and NMO [19, 20].

### Immune cell infiltrates are negligible in the retina during EAE

**Fig. 3:**
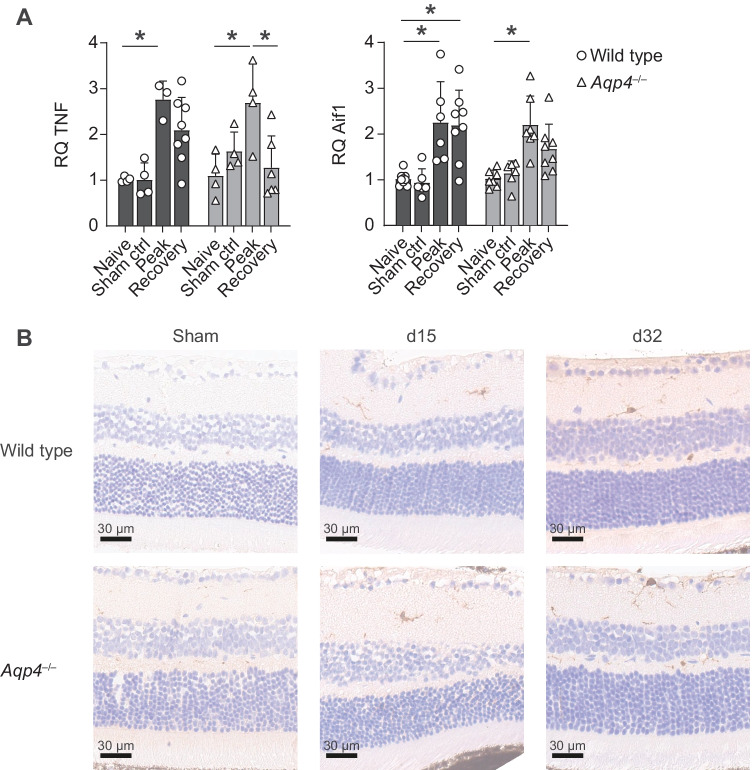
Inflammatory reactions in the retina during EAE. Retinae of PBS/CFA-immunized control mice (sham) as well as of wild-type and *Aqp4*^–/–^ EAE mice were prepared at the indicated time points after immunization (naïve, d15, and d32 p.i.) and either processed for gene expression analysis (**A**) or used for Ionized calcium binding adaptor molecule 1 (Iba1) staining (**B**). **A** Total RNA was isolated from whole retina, transcribed into cDNA and used for quantitative PCR with probes for tumor necrosis factor (TNF), allograft inflammatory factor (Aif-1), and β-actin. Mean relative gene expression (RQ) normalized to naïve wild type controls, RQ ± SD. Two-way ANOVA and Sidak’s post test, * *P* < 0.05. (**C**) Representative Iba1 stainings of the retina (Scale bar, 30 µm)

It was possible that the delayed volume decline of the inner retinal layers in *Aqp4*^–/–^ mice was due to more pronounced cellular inflammation in the retina of *Aqp4*^–/–^ mice as compared to wild-type animals. In order to directly assess this possibility, we characterized the immune cell infiltrate and activation of retinal microglial cells by histology in the visual pathway and the CNS of wild-type and *Aqp4*^–/–^ mice. The retina of either wild-type or *Aqp4*^–/–^ mice did not contain any recruited hematopoietic cells (CD45^+^). Accordingly, we did not detect T cell–derived inflammatory cytokines, such as IFN-γ, IL-17, or GM-CSF, in the retina of either wild-type or *Aqp4*^–/–^ mice during EAE. However, both in wild-type mice and in *Aqp4*^–/–^ mice, we noticed an increased expression of TNF and Iba1 (gene name *Aif1*) in the retina at the peak of EAE as compared to naive or control immunized animals (Fig. 3A). Morphologically, the Iba1-positive cells resembled microglial cells that were detectable in the retinae of wild-type and *Aqp4*^–/–^ mice as of the peak of EAE (Fig. 3B). These data indicated that the retinae of both mouse strains participated in the inflammatory response despite the lack of inflammatory infiltrates from the systemic compartment. This observation was consistent with the idea of waves of resident innate immune cell activation in the retina during EAE that has been previously reported [14]. Notably, a decrease of neurotrophic factors (BDNF and CNTF) was apparent in the retina in the course of EAE, and the expression of both neurotrophic factors was below baseline in the recovery phase – again with similar dynamics in wild-type and *Aqp4*^–/–^ retinae (Supplementary Fig. 2). Together, these data indicated that the retina – despite the absence of hematopoietic inflammatory cells – still showed an inflammatory response, which at the peak of EAE was similar in wild type and *Aqp4*^–/–^ mice. In the CNS (including the optic nerve), we did not detect differences in the amount, spatial distribution, subset composition, or functional phenotype of leukocytes in wild-type and *Aqp4*^–/–^ mice, either (Supplementary Figs. 3 and 4).

### *Aqp4*^–/–^ mice exhibit retinal hyperperfusion at the peak of EAE

**Fig. 4:**
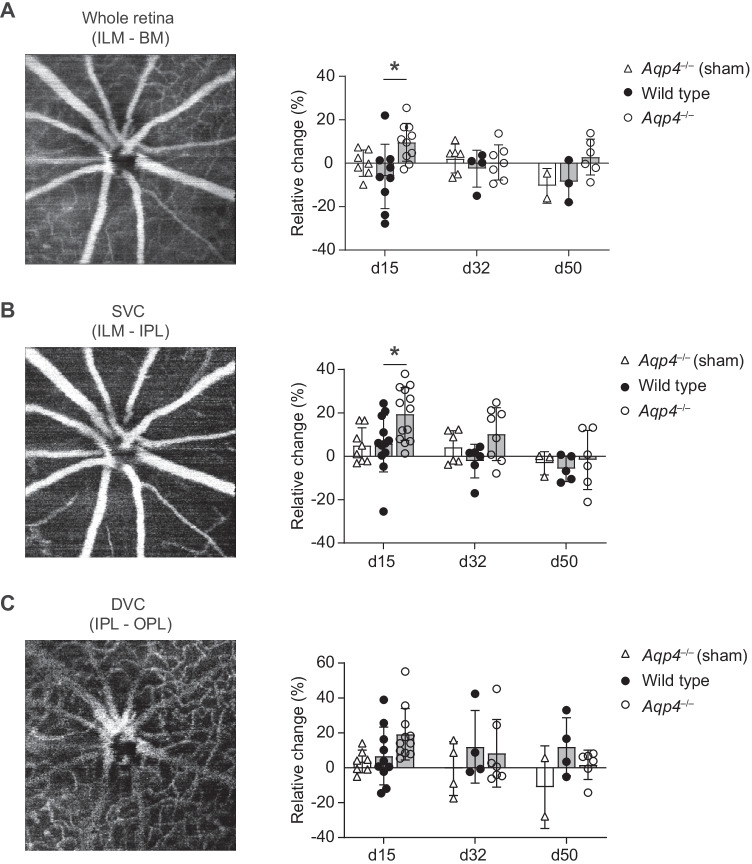
OCT-A identifies vascular dysregulation in the retina in the absence of AQP4. **A** Longitudinal OCT-A measurements were performed in wild-type and *Aqp4*^–/–^ mice prior to immunization (baseline) and on d15 p.i., d32 p.i., and d50 p.i. For OCT-A imaging, consecutive en face images and decorrelation signals were recorded as described in Materials and methods. Segmentation of the ONH area was performed automatically by the in-built software (v2.5.4) into the superficial (SVC) and deep vascular complex (DVC) and corrected manually if necessary in a blinded manner. The vessel density was defined as the proportion of the detected vessel pixels in an extracted OCT-A image with a total number of 512 × 512 pixels and determined using ImageJ software (v.1.53 k). Changes in vessel density in the entire retina (**A**), SVC (**B**), and DVC (**C**) were quantified during EAE as relative change in vessel density with the baseline density (d0) as calibrator (relative change = 1 – (dx/d0)). Mean ± SD. Two-way ANOVA and Sidak’s post test. * *P* < 0.05. *Aqp4*^–/–^ and wild-type mice did not differ in mean vessel densities in all projections at baseline (whole retina: wild-type 0.195 ± 0.023 pixel/area vs. *Aqp4*^–/–^0.194 ± 0.026 pixel/area; SVC: wild-type 0.212 ± 0.029 vs. *Aqp4*^–/–^ 0.206 ± 0.025; DVC: wild-type 0.177 ± 0.023 vs. *Aqp4*^–/–^0.164 ± 0.030). BM, Bruch’s membrane; DVC, deep vascular complex; ILM, inner limiting membrane; IPL, inner plexiform layer; OPL, outer plexiform layer; SVC, superficial vascular complex

Since AQP4 plays an important role in fluid homeostasis in the CNS and is assumed to be relevant in the retina [5], we explored functional and structural changes of the retinal gliovascular junction during EAE. First, longitudinal OCT-A analyses were performed in wild-type and *Aqp4*^–/–^ mice. OCT-A is a novel technology to visualize the capillary network of the retina. Commercial analysis algorithms have only been established for the human retina [21]. Here, we used a human OCT-A segmentation approach allowing us to assess the superficial (SVC – vessels in the RNFL, GCL, and IPL) and the deep (DVC – vessels in the INL and OPL) areas of the retina [21] (Fig. 4A). At the peak of EAE, we identified a larger vessel density in the retinae of *Aqp4*^–/–^ mice as compared to wild-type mice (Fig. 4B), with the SVC contributing more to that increase in the vessel signal of *Aqp4*^–/–^ mice than the DVC (Fig. 4B, C). Therefore, the retinae of *Aqp4*^–/–^ mice appeared to be more densely perfused at the height of inflammation. No difference in the vascular vessel density was found between *Aqp4*^–/–^ mice and control animals at later stages of EAE.

**Fig. 5:**
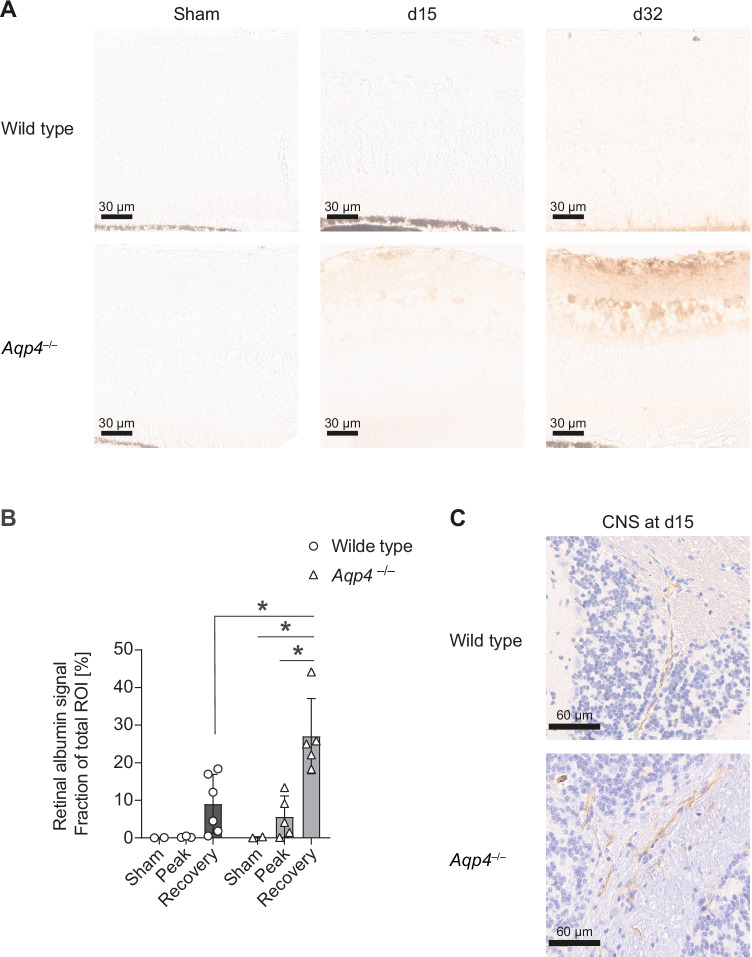
The blood retina barrier (BRB) is disrupted in *Aqp4*^–/–^ mice during EAE. Retinae (**A**) and cerebellar tissue (**C**) were isolated from sham-immunized (PBS/CFA, left) and MOG(35–55)/CFA-immunized wild-type and *Aqp4*^–/–^ mice on d15 (peak) and d32 p.i. (recovery), followed by albumin staining to visualize plasma extravasation (scale bar, 30 µm and 60 µm, counterstain with hematoxylin only in cerebellar sections). **B** Albumin signal was assessed automatically with computer assisted algorithms provided by QuPath v0.3.2 software after annotation of the whole retina (ROI). Albumin signal is shown as mean positive signal area of total ROI in % ± SD. Significance was calculated with two-way ANOVA and Sidak’s post test. * *P* < 0.05

In order to test whether the functional change in the retinal vasculature of *Aqp4*^–/–^ mice coincided with a structural damage of the blood retinal barrier, we performed albumin stainings. We only found slight extravascular albumin deposits in the retinae of wild-type mice in the recovery phase of EAE, and the albumin signal was not significantly different from the retinal albumin signal of sham immunized control mice (Fig. 5A, B). In contrast, extravascular albumin deposits – mostly in inner retinal layers – were pronounced in the retinae of *Aqp4*^–/–^ mice at the peak of EAE and in particular during recovery. The intensity and size of these deposits was significantly greater in the retinae of *Aqp4*^–/–^ mice as compared to sham immunized control animals, and in particular in the recovery stage, the albumin extravasation was increased in *Aqp4*^–/–^ EAE mice as compared with wild-type control EAE mice (Fig. 5A, B). Notably, both strains exhibited albumin deposits in the CNS (as for instance in the cerebellum) that were consistently associated with inflammatory infiltrates (Fig. 5C). Taken together, the lack of AQP4 in the retina resulted in the extravasation of albumin in the absence of inflammatory infiltrates, suggesting that a certain degree of structural damage occurred to the blood retinal barrier of *Aqp4*^–/–^ mice in the course of MOG(35–55)-induced EAE.

#### Lack of AQP4 favors astrogliosis and structural retinal damage in the context of CNS autoimmunity

**Fig. 6:**
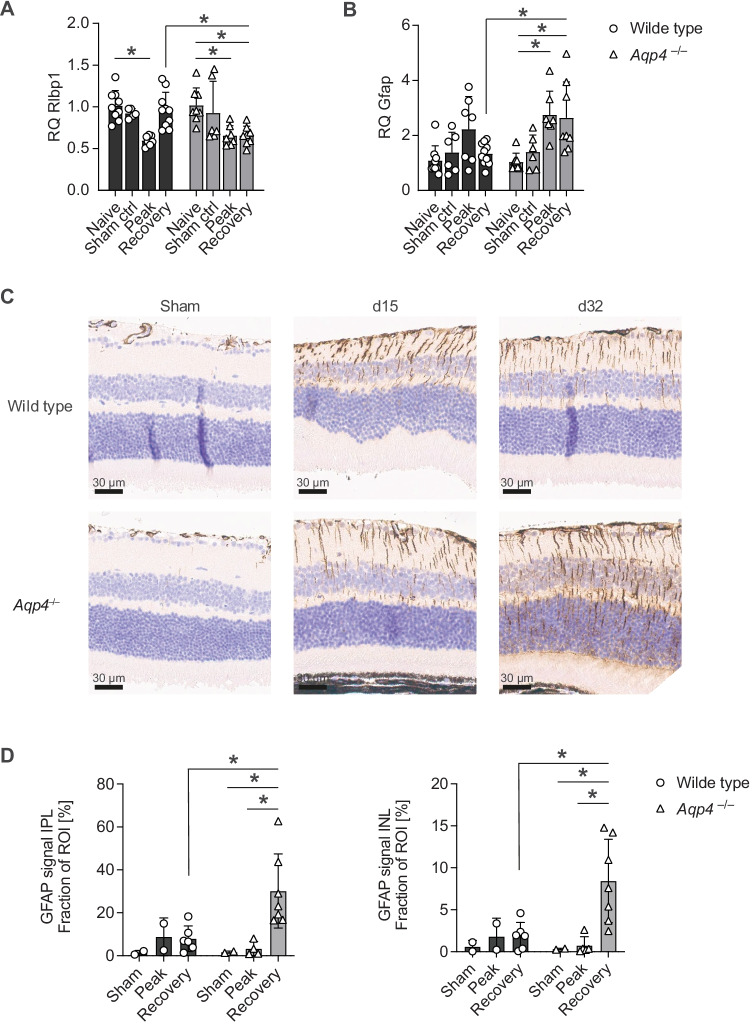
Absence of AQP4 produces signs of Müller cell dysfunction and astrogliosis in the retina during EAE. Retinae of PBS/CFA-immunized control mice (sham) as well as of wild-type and *Aqp4*^–/–^ EAE mice were prepared at the indicated time points after immunization (naïve, d15, and d32 p.i.) and either processed for gene expression analysis (A–B) or used for glial fibrillary acidic protein (GFAP) staining (C). **A, B** Total RNA was isolated from whole retina, transcribed into cDNA and used for quantitative PCR with probes for retinaldehyde-binding protein 1 (Rlbp1), GFAP, and β-actin. Mean relative gene expression (RQ) normalized to naïve wild-type controls, RQ ± SD. **C** Representative GFAP stainings of the retina (scale bar, 30 µm). **D** GFAP signal was assessed automatically with computer assisted algorithms provided by QuPath v0.3.2 software after annotation of IPL and INL (ROI). GFAP signal is shown as mean positive area fraction of total ROI in % ± SD. Significance in A–B and D was calculated with two-way ANOVA and Sidak’s post test. * *P* < 0.05

**Fig. 7:**
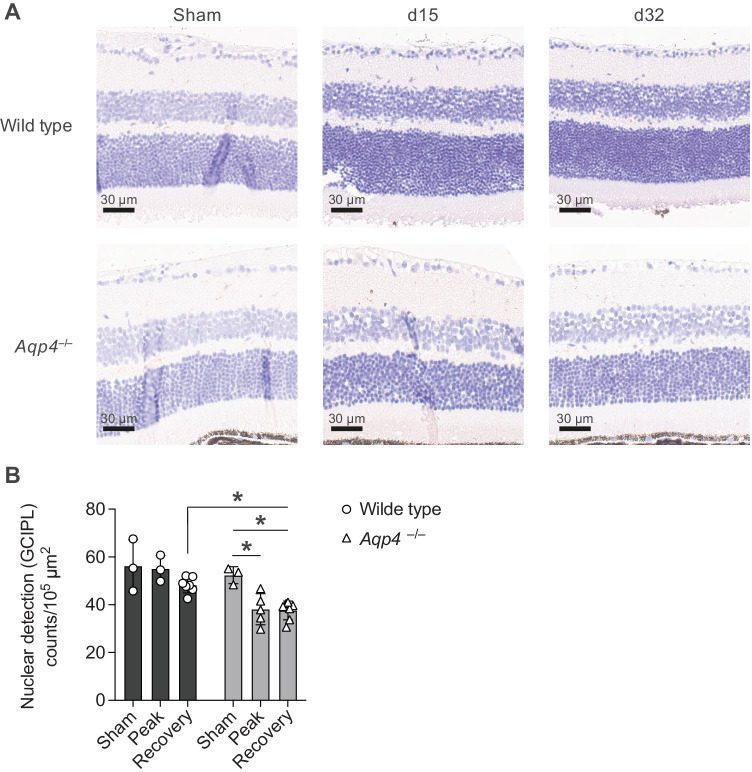
Exaggerated retinal ganglion cell loss in *Aqp4*^–/–^ mice. Retinae of PBS/CFA-immunized control mice (sham) as well as of wild-type and *Aqp4*^–/–^ EAE mice were prepared at the indicated time points after immunization (naïve, d15, and d32 p.i.) and used for CD45 staining (A). **A** Representative CD45 stainings of the retina (scale bar, 30 µm). **B** Nuclei in the ganglion cell layer (GCL) were determined in an automated manner using positive nuclear detection in QuPath (B). Nuclear detection is shown as mean counts per 10^5^ µm^2^ ± SD. Two-way ANOVA and Sidak’s post test, * *P* < 0.05

In order to test tissue specific retinal responses in the CNS of diseased wild-type and *Aqp4*^–/–^ mice, we focused on the assessment of Müller cell integrity by immunohistochemical staining of glutamine synthetase (GS) and by the retinal expression level of retinaldehyde-binding protein 1 (Rlbp1) [9]. While GS expression was not different in wild-type vs *Aqp4*^–/–^ mice (Supplementary Fig. 5), Rlbp1 expression was reduced in the retinae of *Aqp4*^–/–^ mice as compared to wild-type controls during recovery (Fig. 6A), suggesting a direct impact of AQP4-deficiency on Müller cells. Notably, retinal glial fibrillary acidic protein (GFAP) expression in the recovery phase was significantly higher in *Aqp4*^–/–^ mice than in wild-type control EAE mice (Fig. 6B). Importantly, the increased presence of GFAP RNA correlated with a higher protein expression of retinal GFAP protein in the recovery phase in *Aqp4*^–/–^ mice as compared with wild-type mice both in the GCIPL and in the INL (Fig. 6C, D) – a pattern that we found recapitulated also in the optic nerve and the spinal cord (Supplementary Fig. 6). These data suggested that Müller cell dysfunction (as indicated by lower levels of Rlbp1) did not only result in structural damage of the gliovascular unit of the retina but also promoted enhanced scarring processes (GFAP expression) in the retinae of *Aqp4*^–/–^ mice as compared to their wild-type counterparts in the context of sustained CNS inflammation. In order to eventually test whether the enhanced astrogliosis in *Aqp4*^–/–^ mice was associated with structural damage of the retina, we counted the number of ganglion cells in wild-type and AQP4-deficient retinae at different disease stages. While the nuclear count per reference area in the ganglion cell layer (GCL) was unchanged during EAE in wild-type mice, we found a significant reduction of ganglion cells in the retinae of *Aqp4*^–/–^ mice as of the peak of disease (Fig. 7A, B). Notably, during recovery, the number of retinal ganglion cells was significantly smaller in *Aqp4*^–/–^ mice as compared to wild-type control animals (Fig. 7B). In summary, these results indicated that AQP4 was necessary to promote the clearance of inflammatory edema in the retina and – as a consequence – supported the structural integrity of the retinal ganglion cell layer during EAE.

## Discussion

Our study suggests that AQP4 might be instrumental for the efficient re-establishment of homeostatic fluid management in the retina during CNS autoimmunity. While control mice rapidly cleared the increase of retinal thickness after the peak of EAE, *Aqp4*^–/–^ mice exhibited a significantly delayed normalization of retinal swelling after CNS inflammation. Although EAE resulted in the loss of retinal thickness in recovery in control animals (most likely indicating tissue atrophy), almost no reactive astrocytosis was observed in control animals. In contrast, *Aqp4*^–/–^ mice were left with significantly higher expression of GFAP and astrogliosis in their retinae (and the CNS) after clearing of inflammatory infiltrates in the CNS. Importantly, *Aqp4*^–/–^ mice experienced a more severe loss of retinal ganglion cells than wild-type mice in the late stages of EAE. Therefore, functionally intact AQP4 in Müller cells might contribute to retinal resilience during inflammation.

EAE was induced by active immunization with MOG(35–55) in CFA. We did not observe a difference in motor symptoms of wild-type and *Aqp4*^–/–^ mice at the peak of EAE, suggesting that the CD4^+^ T cell-driven immunopathology in the spinal cord and brain was similar in both strains. Accordingly, the amount of infiltrating T cells and myeloid cells as well as the cytokine phenotype of the T cells in the CNS was identical in wild-type and *Aqp4*^–/–^ mice. In a previous report, *Aqp4*^–/–^ mice had been observed to be partly resistant to MOG(35–55) induced EAE [22] although no quantitative analysis of the inflammatory response and in particular the T cell infiltration was performed. Our immunization protocol was similar but led to no qualitative or quantitative difference in the inflammatory response in the CNS of those *Aqp4*^–/–^ mice that developed disease as compared to wild-type mice. The *Aqp4* null allele that had initially been electroporated into CB1-4 ES cells for the generation of knock-out mice [23] was fully backcrossed onto the C57BL/6 J genetic background in our facility. We always used age- and sex-matched C57BL/6 J wild-type control mice that were bred in the same facility but were not in all instances littermates to the *Aqp4*^–/–^ mice. Since EAE is associated with a pronounced vasogenic edema (as a result of the affected blood–brain barrier disruption leading to perivascular fibrin deposition in the CNS) [24, 25], it is to be expected that loss of AQP4 expression in astrocytes might lead to a reduced capacity of the CNS to cope with the pathogenic process as has been observed in other conditions with prominent vasogenic edema [10]. In histologic analyses of MS tissue specimens, increased AQP4 immunoreactivity was found in both active and inactive lesions, suggesting that AQP4 might even be upregulated in response to inflammatory tissue injury [26, 27]. These observations are in line with a potentially worse recovery of *Aqp4*^–/–^ mice from EAE.

Surrogate markers of retinal pathology as assessed by OCT are being developed in MS [1–3, 28]. For instance, an increase in inner retinal layers (specifically INL) has been associated with the extent of tissue inflammation in the CNS and the risk of relapses [29], and successful anti-inflammatory treatment reverses the increased INL thickness [28]. Segmentation of retinal layers in OCT analyses of mice is challenging [30]. However, the retinal thickness correlates with the inflammatory response in the CNS of EAE mice as well [9]. Since we did not detect inflammatory infiltrates in the retinae of either wild-type or *Aqp4*^–/–^ mice, it is possible that remote effects of the inflammatory process in the optic nerve or activation of resident innate immune cells [14] might trigger retinal swelling. The increase in retinal TNF RNA levels during EAE, which has also been shown previously [9], and the detection of Iba1^+^ cells are consistent with this concept and might correspond to the first wave of innate immune activation in the retina during EAE [14] – well before retrograde axonal degeneration occurs. However, we cannot rule out additive effects from both mechanisms. We observed that retinae of *Aqp4*^–/–^ mice experienced a more sustained thickening than wild-type retinae. Therefore, we propose that Müller cells as the major cell type that expresses AQP4 in the retina might be involved in the control of inflammatory tissue responses. In fact impaired clearance of potentially harmful metabolites and insufficient supply of trophic factors by Müller cells have been reported in diabetic retinopathy. As a consequence of this loss-of-function of Müller cells, apoptosis of retinal cells and damage to the gliovascular unit in particular in the INL might occur [31, 32].

Loss of AQP4 has been associated with increased vasogenic edema. Therefore, we aimed at directly visualizing vessel pathology in mice using OCT-A. Here, we found signs of an increased perfusion of inner retinal layers at the peak of disease in *Aqp4*^–/–^ as compared to wild-type EAE mice. Together with retinal microglia and pericytes, astroytic Müller cells shape the blood retinal barrier (BRB). The BRB is tightly regulated especially within inner retinal layers and is also essential for the retinal microvascular integrity [33, 34]. Thus, the increased retinal perfusion as detected in *Aqp4*^–/–^ mice could be the result of an impaired Müller cell function and a dysregulation of the retinal vasculature. Nevertheless, technical limitations concerning the resolution of OCT and OCT-A must be taken into account, and further experiments need to focus on whether the transiently increased perfusion is a compensatory response to an impaired gliovascular junction in the retina of *Aqp4*^–/–^ mice. Although we found signs of structural damage to the BRB in terms of albumin extravasation in *Aqp4*^–/–^, but not wild-type EAE mice, a more detailed structural and ultrastructural investigation of the BRB in *Aqp4*^–/–^ mice is required in order to assess the mechanism how failure of AQP4 expression or function leads to BRB damage under conditions of inflammatory stress.

Retinal ganglion cell loss was more pronounced in *Aqp4*^–/–^ mice than in wild-type control EAE mice, demonstrating an exaggerated structural damage in AQP4-deficient retinae during CNS inflammation that was – due to its time course – most likely not due to transsynaptic degeneration but to direct inflammatory processes in the retina. Recently, reactive retinal astrocytes were shown to directly damage retinal ganglion cells through release of complement component C3 [35]. Other mechanisms of retinal ganglion cell loss during EAE that were directly associated with inflammatory processes in the retina and independent of transsynaptic degeneration and optic neuritis have also been discussed [36–38]. Here, we propose that the failure of AQP4-deficient retinal astrocytes to rapidly clear inflammatory edema may have a detrimental effect for the survival of retinal ganglion cells.

In summary, our study provides evidence for a non-redundant role of AQP4 in retinal integrity in the context of CNS autoimmunity. AQP4 deficiency is associated with pronounced astrogliosis in the CNS including the visual pathway and especially in the retina during CNS autoimmunity. Hence, AQP4 might subserve a role in the management of fluid stress in the retina as a remote result of CNS inflammation. Failure of this compensatory mechanism due to lack of AQP4 might then lead to GFAP expression as a hallmark of scarring and eventually an exaggerated loss of retinal ganglion cells. Overall, our results support the significance of a retinal glymphatic system for interstitial fluid management, which is facilitated by AQP4 expression in Müller cells. This system might even have a universal physiological role also in other retinal disease conditions, such as age-related macular degeneration or glaucoma [39, 40]. The correlation of our findings with visual parameters that require instrument-based quantification [41] will be of particular importance in future studies as well as a closer focus on retinal changes that precede the onset of EAE in order to gain better insight into the initial stages of retinal pathology that unfolds in the absence of inflammatory cell infiltrates. Further, novel tracer-based applications for visualizing glymphatic pathways might help monitor the dynamics of changes in retinal fluid dynamics and their correlation with retinal pathology [40, 42].

## Supplementary Information

Below is the link to the electronic supplementary material.

Supplementary file1 (DOCX 40 KB)

Supplementary file2 (PDF 1713 KB)

## Data Availability

The data that support the findings of this study are available from the corresponding author upon reasonable request.
