# Supplementary material for: Aquaporin-4 prevents exaggerated astrocytosis and structural damage in retinal inflammation
Source: J Mol Med (Berl). 2022 May 10;100(6):933–46. doi: 10.1007/s00109-022-02202-6 (PMC9166880; doi:10.1007/s00109-022-02202-6)
Supplement: Supplementary file 1 — Supplementary file1 (DOCX 40 KB) [file 109_2022_2202_MOESM1_ESM.docx]

**Supplementary Table 1 – Retinal layers during EAE**

| **Thickness (µm)** | **d0** | **d15** | **d32** | **d50** |
| --- | --- | --- | --- | --- |
| **Retina** | | | | |
| WT | 237.39 (± 7.98) | 244.85 (± 5.65) | 233.13 (± 8.86) | 234.29 (± 7.89) |
| *Aqp4*^–/–^ | 233.34 (± 5.43) | 243.23 (± 6.03) | 235.62 (± 5.45) | 232.35 (± 4.68) |
| **RNFL** | | | | |
| WT | 24.01 (± 1.77) | 26.70 (± 3.60) | 20.22 (± 2.88) | 20.69 (± 2.28) |
| *Aqp4*^–/–^ | 22.56 (± 2.5) | 25.90 (± 4.76) | 20.84 (± 1.84) | 20.98 (± 2.48) |
| **GCIPL** | | | | |
| WT | 43.92 (± 3.91) | 45.82 (± 4.02) | 39.31 (± 4.65) | 38.08 (± 3.77) |
| *Aqp4*^–/–^ | 41.97 (± 2.95) | 45.70 (± 4.73) | 39.22 (± 5.35) | 37.08 (± 3.41) |
| **IRL (RNFL + GCIPL)** | | | | |
| WT | 67.93 (± 4.15) | 72.52 (± 2.88) | 59.11 (± 5.87) | 58.77 (± 5.51) |
| *Aqp4*^–/–^ | 64.53 (± 3.23) | 71.60 (± 6.81) | 60.06 (± 5.32) | 58.05 (± 5.57) |
| **INL** | | | | |
| WT | 20.84 (± 0.97) | 22.72 (± 2.33) | 20.86 (± 1.60) | 20.31 (± 1.95) |
| *Aqp4*^–/–^ | 20.63 (± 1.51) | 22.28 (± 1.50) | 21.34 (± 1.43) | 20.01 (± 1.39) |
| **ORL** | | | | |
| WT | 148.61 (± 8.23) | 149.61 (± 5.18) | 152.73 (± 6.16) | 155.21 (± 3.77) |
| *Aqp4*^–/–^ | 148.19 (± 5.33) | 149.35 (± 4.44) | 154.22 (± 5.32) | 154.29 (± 3.98) |

**Retinal layers during EAE.** Longitudinal OCT measurements were performed in wild-type and *Aqp4*^–/–^ mice prior to immunization (baseline), and on d15 p.i., d32 p.i. and d50 p.i.. Layers were segmented automatically with Eye Explorer software and corrected manually in a blinded manner as per APOSTEL 2.0 recommendations [17]. IRL comprises retinal nerve fiber layer (RNFL) and GCIPL. ORL comprises layers between inner and outer limiting membrane. Data are shown as mean absolute thickness in µm ± SD.
