# Supplementary material for: Aquaporin-4 prevents exaggerated astrocytosis and structural damage in retinal inflammation
Source: J Mol Med (Berl). 2022 May 10;100(6):933–46. doi: 10.1007/s00109-022-02202-6 (PMC9166880; doi:10.1007/s00109-022-02202-6)
Supplement: Supplementary file 2 — Supplementary file2 (PDF 1713 KB) [file 109_2022_2202_MOESM2_ESM.pdf]

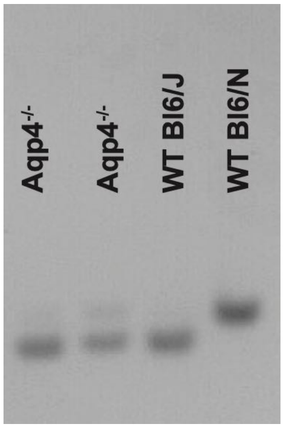

Supplementary Fig. 1 | Lack of Rd8 mutation in *Aqp4*$^{-/-}$ mice. DNA was isolated from ear mark samples of *Aqp4*$^{-/-}$, wild-type C57BL/6J and wild-type C57BL/6N mice and amplified for wild-type and mutant Rd8 allele.

Supplementary Figure 2

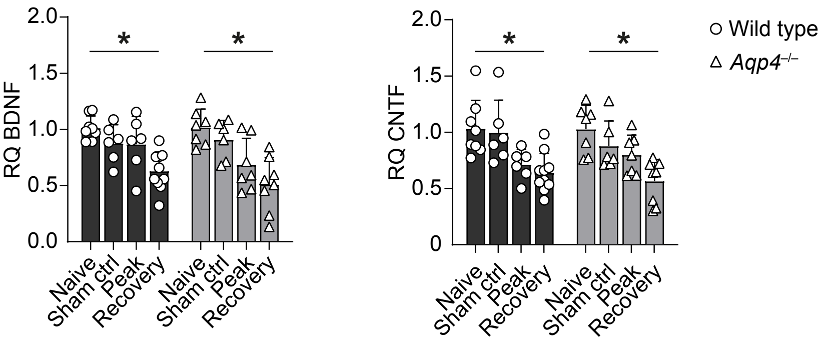

Supplementary Fig. 2 | Gene expression analysis supports the involvement of the retina in the inflammatory response during EAE. Retinae were isolated from PBS/CFA-immunized (sham) and MOG(35-55)/CFA-immunized wild-type and *Aqp4*$^{-/-}$ mice at the peak of EAE and during recovery and used for gene expression analysis. Mean relative gene expression (RQ ± SD) of brain-derived neurotrophic factor (BDNF) and ciliary neurotrophic factor (CNTF) were assessed and internally calibrated to gene expression in unmanipulated (naive) wild-type mice. Two-way ANOVA and Sidak's post test, * $P < 0.05$.

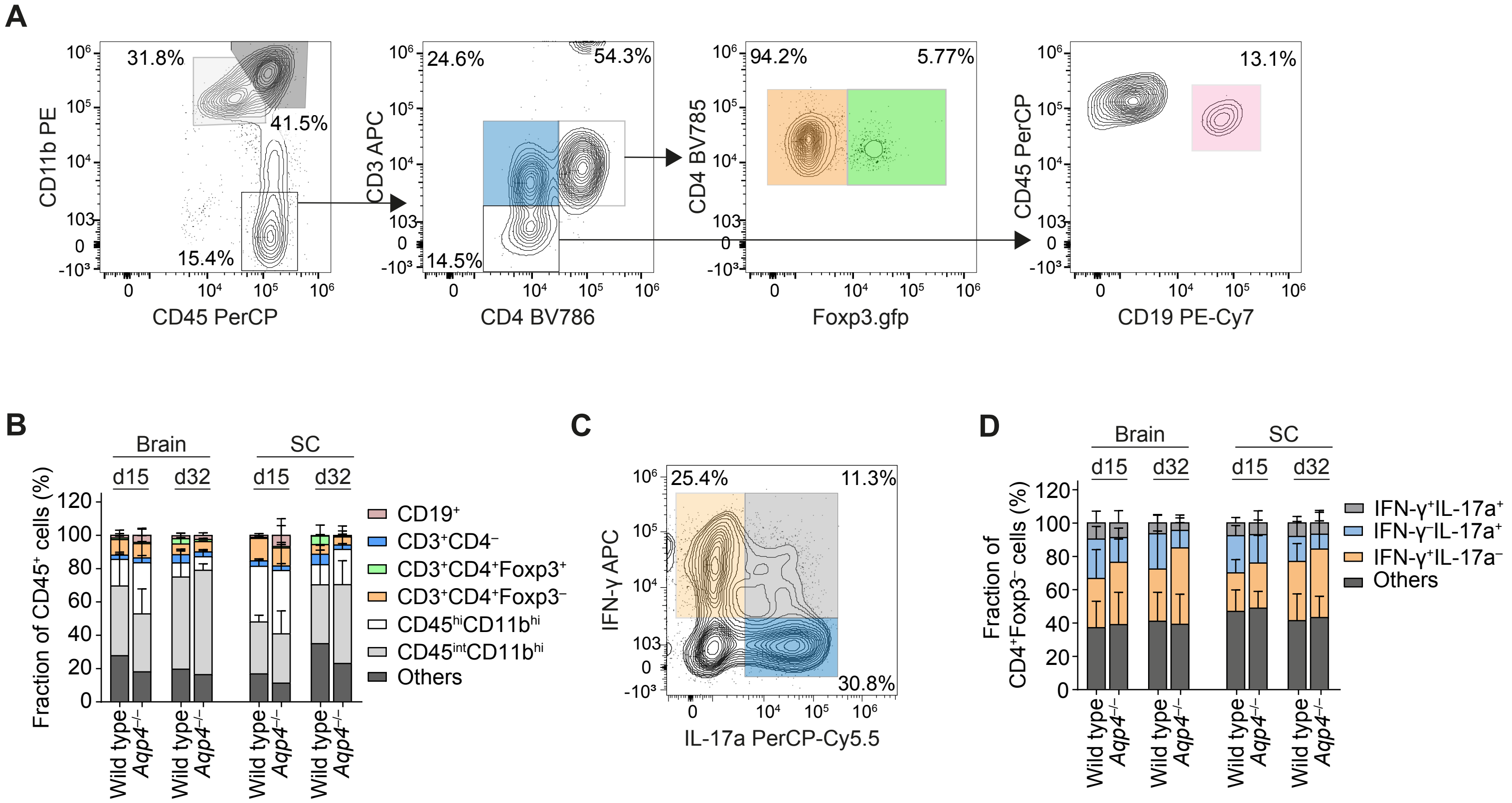

Supplementary Fig. 3 | Flow cytometric characterisation of mononuclear cell infiltrates in the context of CNS autoimmunity supports a similar inflammatory load in wild-type and *Aqp4*$^{-/-}$ mice during EAE. Flow cytometric assessment of mononuclear cell infiltrates and intracellular cytokine staining of CD4^{+} T cells isolated from the CNS of either PBS/CFA- and MOG/CFA-immunized wild-type and *Aqp4*$^{-/-}$ mice at the peak of EAE (d15 p.i.) and during recovery (d32 p.i.). (A) Representative cytograms and gating strategy of mononuclear cell infiltrates isolated from the brain of a MOG/CFA-immunized wild-type mice at peak of EAE (d15 p.i.). (B) Fraction of CD45^{+} cells isolated from brain and spinal cord (SC) at different time points of EAE (d15 p.i. and d32 p.i.). (C) Representative plot of an intracelluar cytokine staining of CD4^{+}Foxp3^{-} T cells isolated from the brain of a MOG/CFA-immunized wild-type mouse at the peak of EAE (d15 p.i.). (D) Fraction of cytokine expressors in CD4^{+}Foxp3^{-} T cells isolated from brain and SC at different time points of EAE of wild-type and *Aqp4*$^{-/-}$ mice (d15 p.i and d32 p.i.).

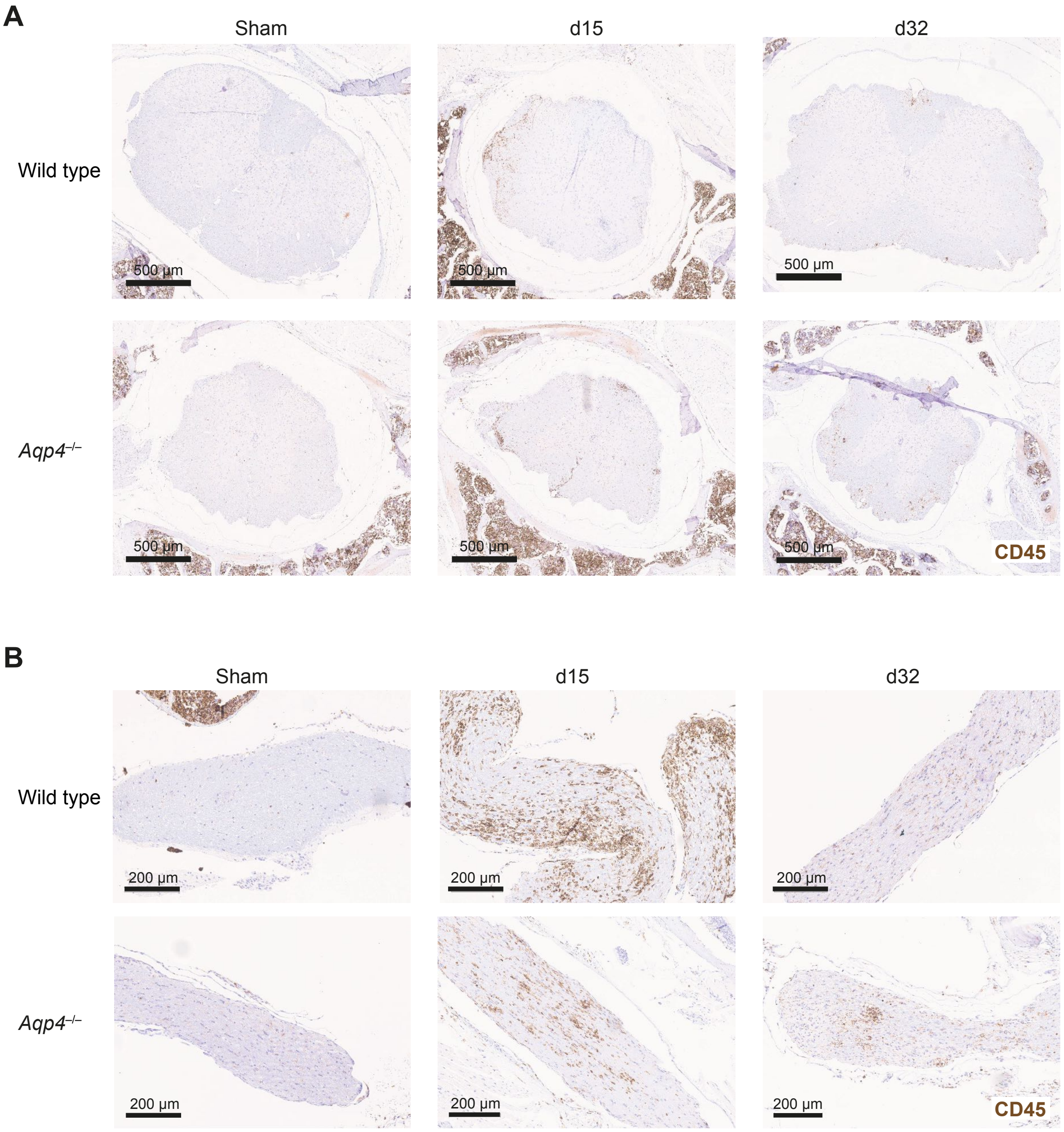

Supplementary Fig. 4 | The amount of mononuclear cell infiltrates in the spinal cord and optic nerve of wild-type and *Aqp4*$^{-/-}$ mice is similar during EAE. CFA-immunized (sham) control mice and wild-type and *Aqp4*$^{-/-}$ mice were prepared on d15 and on d32 p.i. for immunohistochemical assessment of CD45 expression in the spinal cords (A) and the optic nerves (B). Scale bar 500 µm for spinal cord and 200 µm for optic nerves.

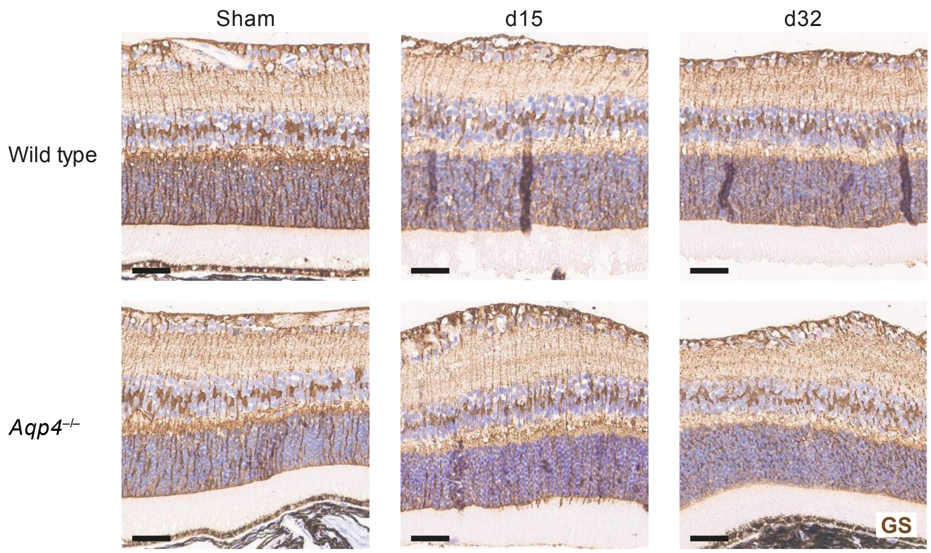

Supplementary Fig. 5 | Glutamine synthetase expression in Müller cells. Retinae of PBS/CFA-immunized control mice (sham) as well as of wild-type and *Aqp4*$^{-/-}$ EAE mice were prepared at the indicated time points after immunization (d15 and d32 p.i.) and used for glutamine synthetase (GS) staining. Note that no gross difference was detected in the retinal GS signal between wild-type and *Aqp4*$^{-/-}$ mice. Representative GS staining of the retina (Scale bar, 30 μm).

Supplementary Figure 6

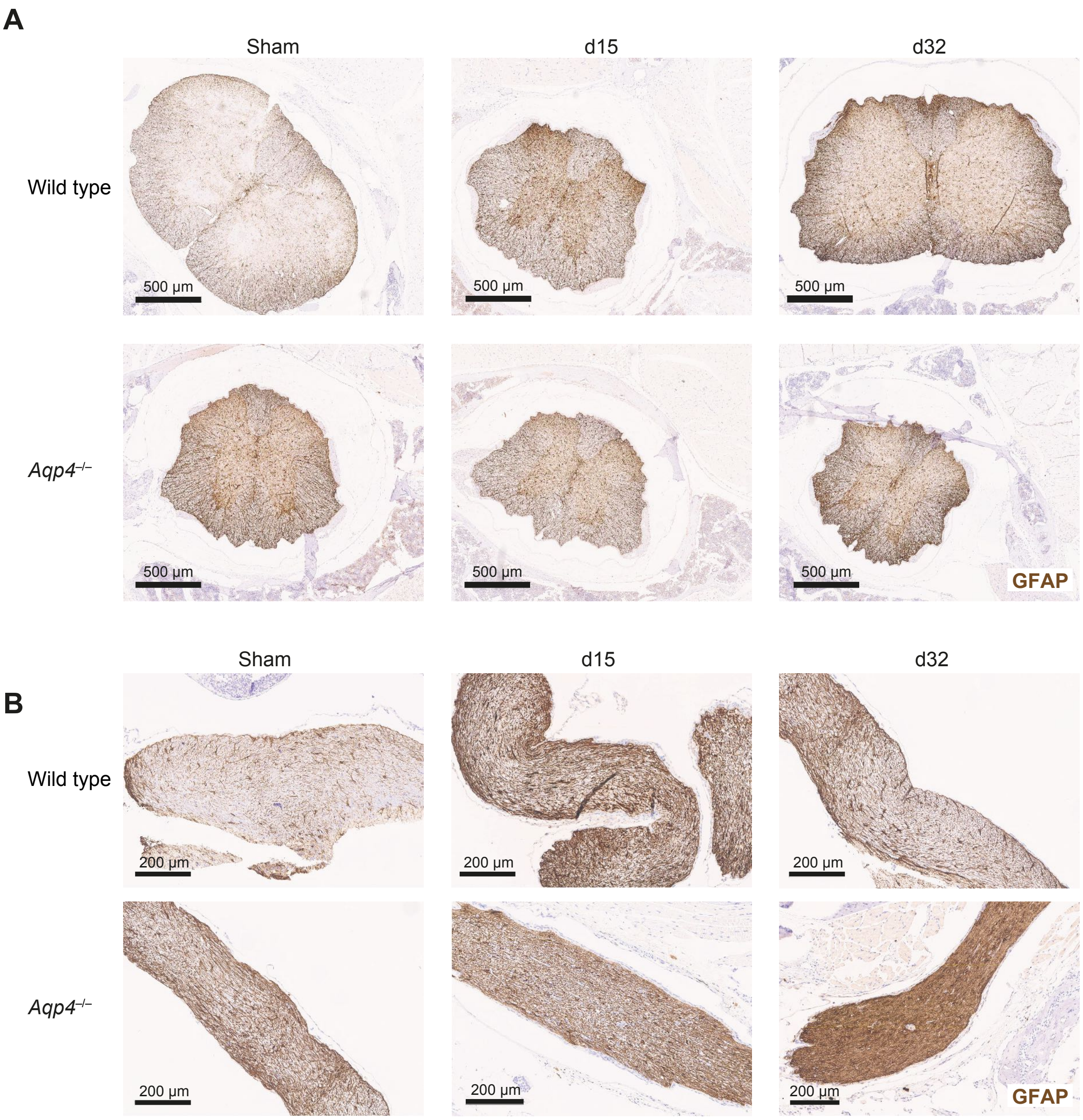

Supplementary Fig. 6 | *Aqp4*$^{-/-}$ mice exhibit persistent astrogliosis during the recovery phase of EAE. MOG(35-55)-immunized wild-type and *Aqp4*$^{-/-}$ mice as well as CFA-immunized control animals (sham) were sacrificed at peak (d15) and recovery (d32) of EAE. (A) Spinal cords and (B) optic nerves were stained for GFAP. Scale bar 500 µm for spinal cord and 200 µm for optic nerves.
